# The role of intraoperative parameters on predicting laparoscopic abdominal surgery associated acute kidney injury

**DOI:** 10.1186/s12882-018-1081-4

**Published:** 2018-10-22

**Authors:** Nattachai Srisawat, Manasnun Kongwibulwut, Passisd Laoveeravat, Nuttha Lumplertgul, Pornlert Chatkaew, Pipat Saeyub, Krittayot Latthaprecha, Sadudee Peerapornratana, Khajohn Tiranathanagul, Somchai Eiam-Ong, Kriang Tungsanga

**Affiliations:** 1 Division of Nephrology, Department of Medicine, Faculty of Medicine, Chulalongkorn University, Rama IV Road, Pathumwan, Bangkok, 10330 Thailand; 2 Excellence Center for Critical Care Nephrology, King Chulalongkorn Memorial Hospital, the Thai Red Cross Society, Bangkok, Thailand; 3 Department of Anesthesiology, Faculty of Medicine, Chulalongkorn University, Bangkok, Thailand

**Keywords:** Acute kidney injury, Laparoscopic abdominal surgery, Intra-abdominal hypertension, Exposure index, NGAL

## Abstract

**Background:**

Laparoscopic abdominal surgery has been widely used to reduce the length of hospital stay and complications from open abdominal surgery. During the operation, the creation of pneumoperitoneum is used for better visualization of the operating field. However, the effect of pneumoperitoneum on kidney function is unknown. We aimed to identify risk factors and predictors associated with AKI development following laparoscopic abdominal surgery.

**Methods:**

A single-center prospective cohort study of laparoscopic abdominal surgery patients between June 2012 and December 2013. Acute kidney injury (AKI) was identified by Kidney Disease Improving Global Outcome (KDIGO) criteria. Urinary neutrophil gelatinase associated lipocalin (uNGAL) was measured on the first 3 days after surgery as a surrogate marker of AKI.

**Results:**

Of the 64 patients, 23 (35%) developed postoperative AKI. The mean age, initial blood pressure, and initial glomerular filtration rate were not different between AKI and non-AKI groups. Inflation time and exposure index were significantly higher in the AKI group compared to non-AKI group (192.0 vs 151.1 min, *p* = 0.045, and 2325.9 vs 1866.1 mmHg-minutes, *p* = 0.035). Operation time, mean intra-abdominal pressure, duration of intraoperative hypotension, amount of blood loss and intravenous fluid were not different between groups. In multivariable analysis adjusted for age, diabetes, baseline estimated glomerular filtration rate, and type of operation (urological surgery), exposure index was significantly associated with postoperative AKI, with odds ratio (95% CI) 1.47 (1.05–2.04), *p* = 0.024. By combining the intraoperative parameters with clinical model the area under the receiver operating characteristic curve was 0.71 (95% CI 0.58–0.84).

**Conclusions:**

AKI was a common condition in laparoscopic abdominal surgery. Exposure index has been proposed as a novel predictor of laparoscopic abdominal surgery associated AKI.

**Electronic supplementary material:**

The online version of this article (10.1186/s12882-018-1081-4) contains supplementary material, which is available to authorized users.

## Background

Laparoscopic abdominal surgery has been widely used in the past few decades. Compared to open surgery, it has various benefits such as small incision, less postoperative pain, reduced length of hospital stay, early mobilization, and shorter recovery period [1–3]. One of the important steps in laparoscopic abdominal surgery is the creation of pneumoperitoneum for better visualization and manipulation of instruments inside the abdominal cavity. Carbon dioxide (CO_2_) is the most common gas insufflated into the peritoneal cavity to create pneumoperitoneum. This key process can induce many physiological changes especially in renal function [3]. In order to prevent complications during intraoperative and postoperative periods it is necessary for physicians to have a good understanding of renal function disturbances induced by the increased intra-abdominal pressure.

In animal experiments, different studies have found conflicting results when identifying physiological changes of renal function from CO_2_-induced pneumoperitoneum [4–7].Chiu and colleagues [4], in a sample of well-hydrated pigs, showed a 60% reduction of superficial renal cortical blood flow after 2 hours of CO_2_ insufflation and, after desufflation, renal blood flow returned to its pre-insufflated state. Further, a pig study from Kirsch and colleagues [8] reported an increase in serum creatinine with decreased urine flow after decreasing inferior vana cava flow in the pneumoperitoneum (15 mmHg). On the other hand, Yavuz and colleagues [6] and Ali and colleagues [7] reported preserved renal blood flow in pigs given more than 15 mmHg pneumoperitoneum. However, a human study to establish the relationship between CO_2_ pneumoperitoneum and renal function is still lacking.

In this study, we aimed to examine the role of intraoperative parameters on predicting acute kidney injury (AKI) following laparoscopic abdominal surgery.

## Methods

### Study population and setting

This was a single-center prospective cohort study performed at King Chulalongkorn Memorial Hospital, Bangkok, Thailand between June 2012 and December 2013.

Eligible participants were adults aged 18 years or older, with American Society of Anesthesiologist (ASA) status between I and III, who underwent laparoscopic abdominal surgery for at least 2 h, long enough to impact kidney function [9]. Exclusion criteria included patients with pre-existing chronic kidney disease (estimated glomerular filtration rate (eGFR) < 60 mL/min/1.73 m^2^) and patients on non-steroidal anti-inflammatory drugs (NSAIDs) 1 week before surgery, because peri-operation kidney function might be affected. All patients received the standard treatment for postoperative care including fluid management and hemodynamic optimization). Every patient received standard dose of inhaled anesthetic agent with the maximum dose at 1.3 minimal alveolar concentration. We used age, dose of nitrous oxide, hemodynamic status, body temperature, and intraoperative opioid dose to adjust the dose of inhaled anesthetic agents. Intraoperative CO_2_ inflation was limited at 15 mmHg.

The study was approved by The Institutional Review Board (IRB No. 393/55), Faculty of Medicine, Chulalongkorn University, Bangkok, Thailand. All participants accepted the protocol, and provided written informed consent.

### Blood and urine samples

Blood samples for measurement of serum creatinine and urine samples for testing urine neutrophil gelatinase associated lipocalin levels (uNGAL) were serially obtained at five time points from the start of surgery: 0, 6, 24, 48 and 72 h. We measured uNGAL using ELISA technique (R&D, Minneapolis, USA). Furthermore, we collected and measured urine output (UO) every 30 min during the operation period, and on days 1, 2 and 3 after operation.

### Clinical data

We collected patient demographic data, pre-operative clinical characteristics, co-morbid conditions (hypertension, diabetes and dyslipidemia) and ASA status. We also collected intraoperative data including type of operation (urology, gynecology, colorectal surgery, upper general abdominal surgery or lower general abdominal surgery), type of inhaled anesthetic agents, operation time, inflation time, intra-abdominal pressure, exposure index, duration of intraoperative hypotension, amount of blood loss, amount of intravenous fluid administered and UO. Operation time was defined as the duration of the procedure from the initiation of skin incision to the completion of skin closure according to anesthesia records. Inflation time was the time after the creation of pneumoperitoneum by CO_2_ inflation. Intra-abdominal pressure was the average pressure after the creation of pneumoperitoneum by CO_2_ inflation. Exposure index, the new parameter, was the product of inflation time and intra-abdominal pressure. Intraoperative hypotension was defined by the mean arterial pressure (MAP) < 65 mmHg during surgery.

### Outcomes ascertainment

AKI was diagnosed using standard Kidney Disease Improving Global Outcomes (KDIGO) criteria [10]. However, due to UO being available only in 24 h increments we had to make the following modifications to the standard UO criteria: ≥ 0.5 ml/kg/h as no AKI; 0.3–0.5 ml/kg/h as stage 2; < 0.3 ml/kg/h as stage 3. Baseline serum creatinine was defined as the most recent pre-hospital creatinine value up to 1 year prior to hospital admission [11, 12].

Body weight on admission was used for calculating the rate of urine flow per kilogram per hour. For AKI staging patients were assigned to their worst KDIGO category according to either serum creatinine or UO.

### Statistical analysis

Statistical analyses were performed using STATA version 15.0, with statistical significance set at *p* < 0.05. Comparisons between groups were performed with Fisher’s Exact test for categorical variables and using Student’s *t*-Test or Kruskal-Wallis one-way analysis of variance by ranks for continuous variables. Categorical data is summarized as counts and percent. Continuous data is summarized as mean (standard deviation) or median (25th, 75th percentile). For comparison of serum creatinine and urine NGAL between AKI and Non-AKI groups over the follow-up times (baseline, 6, 24, 48, 72 h), we used multivariable mixed model adjusted for age, diabetes status, urological surgery status and also the clustering effect from repeated measurements within patients.

We used unadjusted and adjusted logistic regression to individually test the association between the three main exposure variables (operation time, inflation time and exposure index) and AKI development. Risk factors considered for adjustment were priori risk factors including age, diabetes, baseline eGFR and type of operation (urological surgery). For analyses of developing AKI prediction, Area under the receiver operating characteristic (AUC-ROC) curve and reclassification analyses were used to measure the predictive performances of each models. The optimal cutoff points were determined by the largest sum of sensitivity and specificity.

## Results

**Fig. 1:**
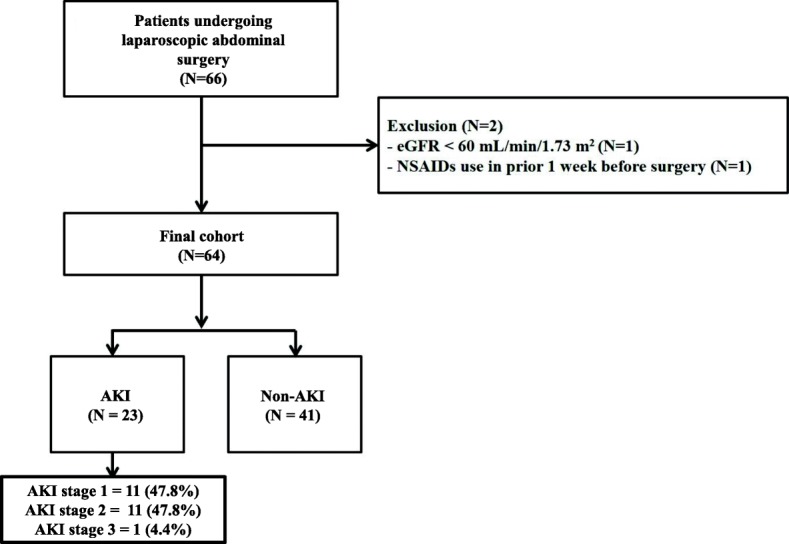
Subject disposition for the study

Sixty six patients who underwent laparoscopic abdominal surgery were screened for the study. Two patients were excluded: one had eGFR < 60 mL/min/1.73 m^2^, and another was on NSAIDs 1 week before surgery. Therefore, 64 patients were included in the study (Fig. 1). Twenty three patients (35.9%) developed postoperative AKI. Eleven patients were diagnosed by creatinine criteria alone and 11 patients were diagnosed by urine output criteria alone. One patient was diagnosed by both criteria (Additional file 1: Table S1). Among AKI patients, 11 had AKI stage 1, 11 patients had AKI stage 2, and 1 had AKI stage 3.

There were no significant differences between the AKI and non-AKI groups in patient demographic or clinical characteristics (Table 1). The age of patients in the AKI and non-AKI groups was not significantly different, with mean of 65 and 61 years, respectively. Baseline serum creatinine, baseline eGFR, co-morbid conditions, baseline vital signs, ASA status, type of operation, and type of inhaled anesthetic agent were similar in both groups (Table 1).

**Table 1:** Patient demographic and clinical characteristics

Characteristics	AKI group 23 (35.9%)	non-AKI group 41 (64.1%)	*P*-value
Age, mean (SD), years	65.1 (11.7)	60.7 (15.3)	0.24
Male, *n* (%)	15 (65.2)	25 (61.0)	0.74
Body weight, median (Q1,Q3), kg	65.7 (59.5,75)	60 (52.8,66.5)	0.20
Height, mean (SD), cm	162.0 (8.3)	160.2 (9.1)	0.43
BMI, mean (SD), kg/m^2^	24.2 (3.6)	24.1 (5.7)	0.94
Baseline serum creatinine, median (Q1,Q3), mg/dL	0.99 (0.79,1.17)	0.85 (0.71,1.13)	0.13
eGFR, mean (SD), mL/min/1.73 m^2^	75.3 (15.5)	85.3 (24.3)	0.08
Heart rate, mean (SD), bpm	73.3 (11.3)	75.5 (15.0)	0.56
Blood pressure. Mean (SD), mmHg			
Systolic	130.0 (17.5)	132.2 (17.8)	0.64
Diastolic	75.6 (12.4)	77.6 (11.7)	0.52
Comorbid conditions			
Diabetes Mellitus, *n* (%)	3 (13.0)	3 (7.3)	0.66^a^
Dyslipidemia, *n* (%)	7 (30.1)	20 (48.8)	0.15
Hypertension, *n* (%)	9 (39.1)	19 (46.3)	0.58
ASA status			0.23^a^
I, *n* (%)	9 (39.1)	8 (19.5)	
II, *n* (%)	11 (47.8)	25 (61.0)	
III, *n* (%)	3 (13.1)	8 (19.5)	
Type of operation			0.39
Urological surgery, *n* (%)	0 (0.0)	1 (2.4)	
Gynecological surgery, *n* (%)	0 (0.0)	3 (7.3)	
Colorectal surgery, n (%)	7 (30.4)	11 (26.8)	
Upper abdomen surgery, *n* (%)	15 (65.2)	26 (63.4)	
Lower abdomen surgery, *n* (%)	1 (4.4)	0 (0.0)	
Inhaled anesthetic agent			
Sevoflurane, *n* (%)	10 (43.5)	12 (29.3)	0.25
Desflurane, *n* (%)	11 (47.8)	27 (65.9)	0.16
Isoflurane, *n* (%)	2 (8.7)	4 (9.8)	> 0.99^a^

*ASA status*, American Society of Anesthesiologists (ASA) status, *BMI* body mass index, *eGFR* estimated glomerular filtration rate, *n* number, *SD* standard deviation, ^a^ Fisher’s exact test

### Surrogate markers of AKI

**Fig. 2:**
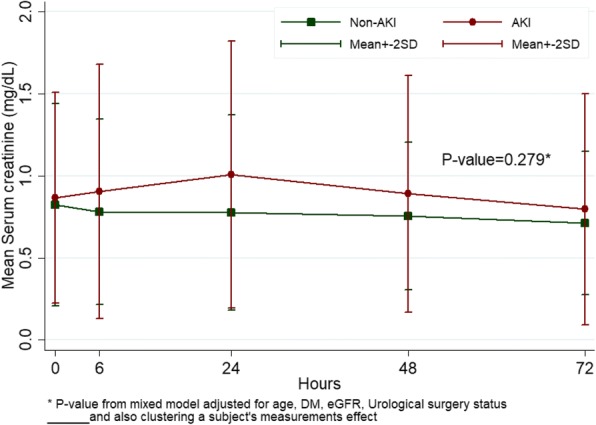
Comparison of serum creatinine at different time points between AKI and non-AKI groups

**Fig. 3:**
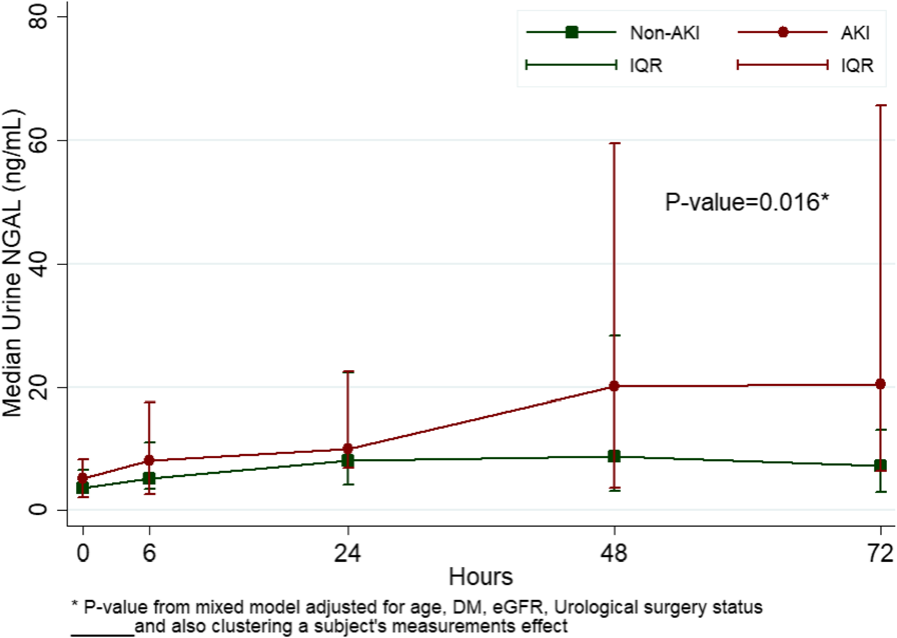
Comparison of uNGAL at different time points between AKI and non-AKI groups

The peak serum creatinine was seen 24 h after surgery with a significant difference between AKI and non-AKI patients: mean (SD) 1.01 (0.41) vs 0.78 (0.30) mg/dL, *p* < 0.013 (Fig. 2). Serum creatinine returned to baseline on postoperative day 3 in both groups. The median uNGAL level of AKI patients increased from 5.1 ng/mL at time 0 to 20.4 ng/mL 72 h after surgery. There median uNGAL level was significantly higher in the AKI group than in the non-AKI group at 72 h: median (Q1,Q3), 20.4 (6.4, 65.5) vs. 7.2 (3.0, 13.2) ng/mL, *p* = 0.037. By performing a paired time-series analysis, uNGAL level in the AKI group was higher than the non-AKI group, *p* = 0.016 (Fig. 3).

### Intraoperative parameters stratified by AKI status

**Fig. 4:**
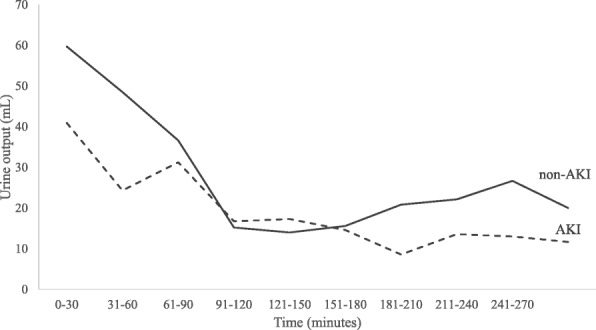
Urine output in 30 min-period during the operation time: AKI group (long dotted line) and non-AKI group (black solid line)

As shown in Table 2, the mean inflation time was significantly higher in the AKI group than in the non- AKI group: mean (SD) 192 (86.4) vs 151.1 (70.7) min, *p* = 0.045. Similarly, mean exposure index was significantly higher in AKI as compared to non-AKI patients: 2325.9 (1083.5) vs. 1806.1 (827.7) mmHg-min, *p* = 0.035. However, intra-abdominal pressure was not different between groups. In addition, duration of intraoperative hypotension, amount of blood loss, intravenous fluid and intraoperative urine output were not different between groups (Fig. 4).

**Table 2:** Intraoperative parameters stratified by AKI status

Parameters	AKI group (*n* = 23)	non-AKI group (*n* = 41)	*P*-value
Operation time, mean (SD), min	299.3 (110.3)	253.0 (100.2)	0.09
Inflation time, mean (SD), min	192.0 (86.4)	151.1 (70.7)	0.045
Exposure index, mean (SD), mmHg-min	2325.9 (1083.5)	1806.1 (827.7)	0.035
Mean intra-abdominal pressure, mean (SD), mmHg	12.1 (2.0)	12.1 (1.9)	0.96
Duration of intraoperative hypotension, median (Q1,Q3), min	1.0 (0,5)	01 (0,10)	0.89
Amount of blood loss, median (Q1,Q3), mL	100 (50,270)	100 (50,400)	0.75
Amount of intravenous fluid, mean (SD), mL	1789 (800)	2172 (1007)	0.12

Exposure index, inflation time by mean of intra-abdominal pressure; Intraoperative hypotension: MAP < 65 mmHg; *n* number, *SD* standard deviation; intraoperative hypotension was defined as mean arterial pressure less than 65 mmHg

### Intraoperative parameters and predicting laparoscopic abdominal surgery associated AKI

Table 3 listed the derived sensitivities, specificities, and predictive values for operation time, inflation time, and exposure index at the cutoff that provided the maximum summation of sensitivity and specificity. The sensitivity and specificity of operation time, inflation time, and exposure index were optimal at the 255 min cutoff with AUC 0.61 (95% CI) 0.46–0.75; 150 min cutoff with AUC 0.63 (95% CI 0.49–0.78); and 1950 mmHg-mim with AUC 0.64 (95% CI 0.49–0.80), respectively.

**Table 3:** The cutoff of intraoperative parameters to predict AKI

Intraoperative parameters	AUC (95% CI)	Cutoff	Sensitivity	Specificity	PPV	NPV
Operative time, min	0.61 (0.46–0.75)	255	60.9%	56.1%	43.8%	71.9%
Inflation time, min	0.63 (0.49–0.78)	150	69.6%	48.8%	43.2%	74.1%
Exposure index, mmHg-min	0.64 (0.49–0.80)	1950	60.9%	56.1%	43.8%	71.9%

*AUC* area under curve, *PPV* positive predictive value, *NPV* negative predictive value, min, minute

**Fig. 5:**
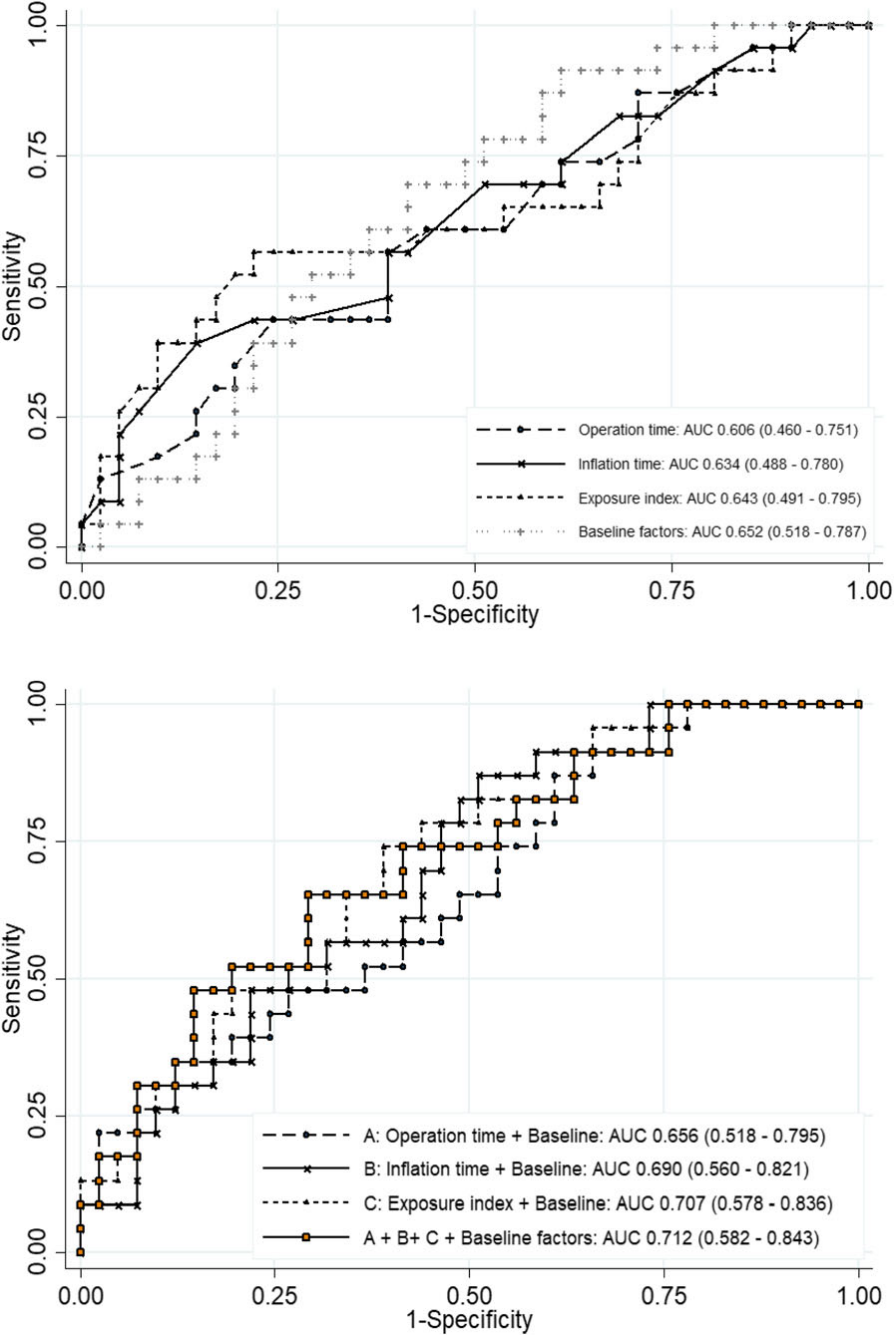
The area under the curve (AUC) for predictive factors of AKI after laparoscopic abdominal surgery. **a** operation time alone, inflation time alone, exposure index alone, and baseline factors alone*. **b** operation time + baseline factors* (**a**), inflation time + baseline factors* (**b**), exposure + baseline factors* **c**). operation time + inflation time + exposure index + baseline factors*.* baseline factors including age, diabetic status, baseline eGFR, and type of operation (urological surgery).

In the multivariate model, adjusted for age, diabetic status, baseline eGFR, and type of operation (urological surgery), exposure index was the risk factors of AKI development with the OR of 1.47 per 500 exposure index increment, 95% CI 1.05–2.04, *p* = 0.024. Whereas inflation time had marginally significant association with the development of AKI, with OR of 1.24 per 30 min increment, 95% CI 1.01–1.58, *p* = 0.048 (Table 4). The AUC-ROC curve of operation time, inflation time, and exposure index for predicting postoperative AKI were 0.61, 0.63, and 0.64, respectively (Fig. 5a). While the model combining of intraoperative parameters with adjusted factor showed the AUC-ROC were 0.66 (operation time with adjusted factors), 0.69 (inflation time with adjusted factor), 0.71 (exposure time with adjusted factors), and 0.71 (operation time + inflation time + exposure time + adjusted factors) (Fig. 5b).

**Table 4:** Analysis of intraoperative parameters to predict laparoscopic abdominal surgery associated AKI

Predictors	Odds ratio (95% CI)	*P*-value	Adjusted Odds ratio^a^ (95% CI)	*P*-value
Operation time (30 min)	1.14 (0.98–1.32)	0.096	1.15 (0.98–1.36)	0.087
Inflation time (30 min)	1.23 (1.00–1.5)	0.051	1.24 (1.01–1.58)	0.048
Exposure index (500 mmHg-min)	1.35 (1.01–1.8)	0.042	1.4 (1.05–2.04)	0.024

^a^Adjusted for age, diabetic status, baseline eGFR, type of operation (urological surgery)

*AKI* acute kidney injury, *CI* confidence interval

### Reclassification of AKI by intraoperative parameters

We also assessed the capability of intraoperative parameters (to ‘reclassify’ the degree of risk of AKI. Based on the clinical prediction model (operation time, inflation time, and exposure index), subjects were categorized into pre-specified ‘low’, ‘intermediate’, and ‘high’ risk of AKI (Table 5) using cutoffs of 0–30%, 30 to 60%, and **>** 60%, respectively. We then compared the proportions of reclassified subjects across each of these three risk groups when intraoperative parameters was added to the clinical prediction model. Among 23 patients who had AKI, 26.1% were reclassified as increased risk of when intraoperative parameters was added to the clinical prediction model and 22.0% were reclassified as lower risk. Among 41 non-AKI patients, 12.2% were reclassified as increased risk for AKI whereas 22% were reclassified as lower risk for AKI. There was no overall significant improvement in reclassification among AKI and non-AKI when intraoperative parameters were combined with clinical model (net reclassification index (NRI) 14.1%, *p* = 0.41). However, using the relative IDI, the reclassification of risk of AKI improved by 1.8% when intraoperative parameters were combined with clinical model (*p* = 0.025).

**Table 5:** Risk reclassification using intraoperative parameters and clinical predictors compared with clinical predictors alone

Model with clinical predictors alone^a^	Model with clinical predictors + intraoperative parameters^b^			Direction of reclassification	
				Increase risk	Decrease risk
Non-AKI (*N* = 41)	< 30% risk	30–60% risk	> 60% risk		
< 30% risk	12 (75)	4 (25)	0		
30–60% risk	9 (37.5)	14 (58.3)	1 (4.2)	5 (12.2)	9 (22.0)
> 60% risk	0	0	1		
AKI (*N* = 23)					
< 30% risk	1	3	0		
30–60% risk	5 (27.8)	10 (55.6)	3 (16.7)	6 (26.1)	5 (22.0)
> 60% risk	0	0	1		

^a^Clinical predictors alone including age, diabetic status, eGFR and urological surgery status

^b^Intraoperative parameters including operation time, inflation time and exposure index

Data in shaded boxes (diagonals) represent similar risk classification between clinical model and clinical plus intraoperative parameters

Numbers to the right of diagonals represent the patients who were reclassified as increased risk by adding intraoperative parameters to the clinical model. Numbers to the left of the diagonals represent patients who were reclassified as lower risk when intraoperative parameters are added to the clinical model

Parentheses represent the percentage of reclassification in each risk category. The net reclassification improvement (NRI) for Non-AKI and AKI was calculated from the difference in proportions moving up and down among AKI and Non-AKI (NRI = 14.1% (95% confidence interval 0.0 to 48.6%, *P* = 0.409)). The relative integrated discrimination improvement (IDI) was measured for the increment in the predicted probabilities for the subset experiencing Non-AKI and the decrement for the subset experiencing AKI (relative IDI = 1.8% (95% CI 1.1 to 16.3%, *P* = 0.025))

## Discussion

The present single-center prospective cohort study in patients who underwent laparoscopic abdominal surgery shows that AKI is a common condition. This study demonstrated the association between inflation time and exposure index with AKI following laparoscopic abdominal surgery. Our results indicate that high exposure index is a significant predictor for AKI in this setting.

The incidence of AKI in our study was 35.9% which is higher than in previous studies. For example, Sharma et al.[9] and Abdullah et al.[13] found that the incidence of AKI following laparoscopic bariatric surgery in morbidly obese patients ranged from 2.3 to 2.9%. However, both studies did not use urine output as diagnostic criteria. We used standard KDIGO criteria, taking into account of both serum creatinine and urine output criteria which possibly allowed for broader AKI detection. Furthermore, the two previous studies only included patients with laparoscopic bariatric surgery and different types of laparoscopic abdominal surgery might have different AKI rates. Of note, the amount of intravenous fluid during operation in non-AKI is higher than AKI group. However, due to the small sample size, this difference is not reach statistical significance. It should be remarked that intravenous fluid might lower the serum creatinine level and misdiagnosed AKI.

Our study proposed a new parameter “exposure index” which aimed to emphasize the interaction between CO_2_ inflation time and intra-abdominal pressure in the occurrence of oliguria and AKI. Interestingly, prolonged time of CO_2_ inflation shown as exposure index and CO_2_ inflation time alone were associated with AKI (Tables 2, 3). However, intra-abdominal pressure alone was not different between the AKI and non-AKI groups which might be explained by better control of intra-abdominal pressure during the creation of pneumoperitoneum. The mean intra-abdominal pressure in our study was 12 mmHg in both groups. This finding corresponds to previous studies in pigs which show decreased renal function when intra-abdominal pressure had to be higher than 20 mmHg [14]. Despite the fact that mean intra-abdominal pressure of 12 mmHg had less effect to perioperative AKI, our findings still emphasize that surgeons and anesthesiologists should avoid prolonged periods of CO_2_ inflation by limiting the inflation time and exposure index in order to reduce the risk of perioperative AKI.

In our study, operation time was not associated with AKI following laparoscopic abdominal surgery. This differed from previous study which found that operation time of more than 210 min increased the risk of AKI from 0.8 to 4.4% (Tables 2, 3) [9].

Several studies have proposed different mechanisms of renal dysfunction during increased intra-abdominal pressure [8]. Theoretically, there are three different mechanisms. The first mechanism, direct chemical effect, is the result of hypercarbia induced by CO_2_ insufflation. The second mechanism is a mechanical effect which relates to increased intra-abdominal pressure [3, 15]. In animal models, when intra-abdominal pressure was more than 20 mmHg, renal vessels were compressed and lead to significant reduction of renal blood flow [14]. This mechanism could not explain the finding in our study because all patients had mean intraabdominal pressure at 12 mmHg. Lastly, renin-angiotensin-aldosterone system (RAAS) might contribute to the mechanism of pneumoperitoneum induced renal dysfunction. This is supported by evidence of renal vasoconstriction at the renal cortex and increase in renin, aldosterone and antidiuretic hormone during laparoscopic gastric bypass surgery [16].

We used uNGAL, a novel surrogate biomarker for AKI, and found that most AKI patients had uNGAL levels below 100 ng/mL, which indicates that mild AKI severity could have played a major role in this group of patients [17]. Currently, there is no standard cut-off level for uNGAL to diagnose AKI but a previous study [18] proposed to define the severity of tubular epithelial cell damage by using the change in biomarker concentration from baseline. The study also showed that an increase in biomarker was associated with higher mortality. In our study, on post-operative day 3, the levels of uNGAL in the AKI group had increased approximately 4 times from baseline (Fig. 3) while serum creatinine returned to baseline (Fig. 2), which indicates that patients experienced “transient AKI”. In some high risk AKI settings, such as myocardial infarction and post-operative vascular surgery, patients with transient AKI had increased mortality by 1.7 and 2.1 times, respectively [19, 20].

Overall, in our study the predictive value of intraoperative parameters did not improve AKI prediction when it was combined with the clinical model. However, using IDI, the reclassification of risk of AKI improved by 1.8% when intraoperative parameters were combined with the clinical model (Table 5).

Our study has several strengths. First, we explored the role of various intraoperative parameters in predicting laparoscopic abdominal surgery associated AKI. Second, we used the combined urine output and serum creatinine criteria to diagnose AKI which could capture more AKI patients than in previous studies. Third, patients receiving potential insults to the kidney (NSAIDs) and patients with chronic kidney disease were excluded thus better isolating the effect of pneumoperitoneum on AKI occurrence.

Admittedly, our study has some limitations. First, the study design was as a single-center prospective cohort study and generalizing the findings would require validation in a larger study. Second, the sample size was rather small. Therefore, the recommendation for further studies in larger populations is still warrant. Third, for an adjusted logistic regression to test the association between the three main exposure variables (operation time, inflation time and exposure index) and AKI development, we chose the priori risk factors for adjustment including age, diabetes, baseline eGFR and type of operation (urological surgery), not from the univariate analysis .

## Conclusions

AKI following laparoscopic abdominal surgery was a common condition which could occur transiently. By limiting the inflation time and exposure index perioperative renal dysfunction could be reduced. In our study, exposure index has been proposed as a novel predictor of laparoscopic abdominal surgery associated AKI. However, a large randomized controlled trial is needed to prove this concept.

## Additional file

Additional file 1: **Table S1.** Diagnostic criteria for AKI in the AKI patients. (DOCX 18 kb)

## Abbreviations

AKI: Acute kidney injury
ASA status: American Society of Anesthesiologists (ASA) status
AUC-ROC: Area under the receiver operating characteristic
BMI: Body mass index
CO_2_: Carbon dioxide
CI: Confidence interval
eGFR: Estimated glomerular filtration rate
KDIGO: Kidney Disease Improving Global Outcomes
NSAIDs: (non-steroidal anti-inflammatory drugs)
RAAS: Renin angiotensin aldosterone system.
SD: Standard deviation
uNGAL: Urine neutrophil gelatinase associated lipocalin
