# Supplementary material for: The role of intraoperative parameters on predicting laparoscopic abdominal surgery associated acute kidney injury
Source: BMC Nephrol. 2018 Oct 22;19:289. doi: 10.1186/s12882-018-1081-4 (PMC6198484; doi:10.1186/s12882-018-1081-4)
Supplement: Supplementary file 1 — Table S1. Diagnostic criteria for AKI in the AKI patients. (DOCX 18 kb) [file 12882_2018_1081_MOESM1_ESM.docx]

**Supplementary Table 1**. **Diagnostic criteria for AKI in the AKI patients**

| **No** | **Baseline sCr** | **sCr day 0** | **sCr Hour 6** | **sCr day 1** | **sCr day 2** | **sCr day 3** | **UO day 1  (mL/kg/hour)** | **UO day 2 (mL/kg/hour)** | **UO day 3 (mL/kg/hour)** | **AKI diagnosed**  **by sCR** | **AKI**  **diagnosed**  **by UO** |
| --- | --- | --- | --- | --- | --- | --- | --- | --- | --- | --- | --- |
| 1 | 1.6 | 1.3 | 1.6 | 1.7 | 1.8 | 1.5 | 0.56 | 0.83 | 1.30 | 1 | 0 |
| 2 | 1.4 | 1.4 | 0.9 | 1.6 | NA | NA | 1.07 | 1.64 | 3.44 | 1 | 0 |
| 3 | 1.2 | 1.3 | 1.4 | 1.6 | 1.5 | 1.3 | 1.26 | 3.79 | 3.16 | 1 | 0 |
| 4 | 2.1 | 1.3 | 1.5 | 1.6 | 1.4 | 1.4 | 0.98 | 2.93 | 2.76 | 1 | 0 |
| 5 | 1.0 | 0.8 | 1.1 | 1.1 | 0.8 | NA | 0.72 | 4.48 | NA | 1 | 0 |
| 6 | 1.2 | 0.8 | 1.2 | 1.1 | 1.1 | 0.9 | 1.77 | 1.67 | 1.40 | 1 | 0 |
| 7 | 0.9 | NA | 0.6 | 0.9 | 0.9 | 0.7 | 1.16 | 2.25 | 2.37 | 1 | 0 |
| 8 | 1.1 | 0.9 | 0.9 | 1.5 | 1.1 | 0.9 | 0.61 | 2.22 | 3.16 | 1 | 0 |
| 9 | 0.9 | 1.0 | 1.8 | 0.8 | 0.8 | 0.8 | 0.82 | 1.79 | 3.16 | 1 | 0 |
| 10 | 1.1 | 0.8 | 1.1 | 1.1 | 0.9 | 0.8 | 0.61 | 0.59 | 1.57 | 1 | 0 |
| 11 | 0.9 | 0.8 | 0.5 | 1.0 | 1.0 | 0.8 | 0.55 | 1.22 | 2.15 | 1 | 0 |
| 12 | 0.9 | 0.5 | 1.0 | 0.9 | 0.8 | NA | 0.49 | 1.51 | 2.79 | 1 | 1 |
| 13 | 1.3 | 1.2 | 1.2 | 1.3 | 0.9 | 1.0 | 0.46 | 1.78 | 2.14 | 0 | 1 |
| 14 | 0.7 | 0.5 | 0.4 | 0.4 | 0.5 | 0.5 | 0.47 | 1.51 | 1.18 | 0 | 1 |
| 15 | 1.2 | 1.2 | 1.0 | 1.0 | 0.8 | 0.8 | 0.30 | 1.29 | 1.14 | 0 | 1 |
| 16 | 0.7 | 0.7 | 0.7 | 0.8 | 0.7 | NA | 0.09 | 3.16 | 1.58 | 0 | 1 |
| 17 | 1.1 | 1.0 | 1.1 | 1.1 | 1.0 | 0.8 | 0.45 | 2.56 | 1.07 | 0 | 1 |
| 18 | 0.8 | 0.7 | 0.7 | 0.8 | 0.7 | 0.7 | 0.47 | 0.79 | 2.37 | 0 | 1 |
| 19 | 0.7 | 0.6 | 0.3 | 0.5 | 0.5 | 0.5 | 0.46 | 1.23 | 1.51 | 0 | 1 |
| 20 | 0.6 | 0.5 | NA | 0.5 | 0.4 | 0.4 | 0.47 | 0.58 | 2.57 | 0 | 1 |
| 21 | 0.7 | 0.6 | 0.5 | 0.5 | NA | NA | 0.32 | 1.26 | NA | 0 | 1 |
| 22 | 0.9 | 0.4 | 0.4 | 0.5 | 0.4 | 0.6 | 0.48 | 1.26 | 2.69 | 0 | 1 |
| 23 | 0.9 | NA | NA | NA | NA | NA | 0.33 | 0.94 | 0.72 | NA | 1 |

AKI, acute kidney injury; UO, urine output; sCr, serum creatinine
